# Long-term results of surgical angioplasty for left main coronary artery stenosis: 18-year follow-up

**DOI:** 10.1186/s13019-015-0209-x

**Published:** 2015-01-17

**Authors:** Jae Han Jeong, Won Yong Lee, Eung-Joong Kim, Sung Woo Cho, Kun Il Kim, Hyoung Soo Kim

**Affiliations:** 1 Department of Cardiothoracic Surgery, Hallym University Sacred Heart Hospital, 896, Pyeongchon-dong, Dongan-gu, Anyang-si, Gyeonggi-do 431-796 South Korea; 2 Department of Cardiothoracic Surgery, Hallym University Dongtan Sacred Heart Hospital, Seoku-dong, Hwaseong-si, Gyeonggi-do 445-907 South Korea; 3 Department of Cardiothoracic Surgery, Hallym University Gangdong Sacred Heart Hospital, Gil-dong, Gangdong-gu, Seoul, 134-701 South Korea

**Keywords:** Coronary artery disease, Coronary artery bypass, Angioplasty, Pericardium

## Abstract

**Background:**

The aim of this study was to determine the long-term outcomes of surgical angioplasty for left main coronary artery (SA-LMCA) stenosis.

**Methods:**

We retrospectively analyzed data from 24 consecutive patients (mean age, 55 years; male/female, 12/12) who underwent a surgical angioplasty for the left main coronary artery (LMCA) stenosis at our institution between 1995 and 2002. We used autologous pericardium in 7 patients and bovine pericardium in 17 patients as a patch. We evaluated the late mortality and major adverse cardiac events (MACE) rate.

**Results:**

There was no operative mortality. Control coronary angiography exhibited wide open and funnel-shaped LMCA in all patients. One patient was lost to follow-up. During the mean follow-up of 167 months, there were 3 sudden cardiac deaths, 4 non-cardiac related deaths, and 9 MACE with one death at reoperation. The Kaplan-Meier method identified freedom from cardiac death in 95.7, 87.0, and 82.4% of the patients, and freedom from MACE in 91.3, 69.6, and 57.7% of the patients at 5, 10, and 15 years, respectively.

**Conclusions:**

This study demonstrated that the long-term outcomes of SA-LMCA with a pericardial patch are acceptable compared to those of coronary artery bypass grafting, despite the controversy over the indications and the patch material used.

## Background

Surgical angioplasty of the left main coronary artery (SA-LMCA) was introduced in 1965 by Effler and Sabiston, and revived by Hitchcock et al. after 20 years [1-3]. Dion, Sullivan, Villemot, and coworkers refined the surgical approach and technique and contributed to its widespread use [4-6]. Despite its inherent advantage of restoring natural antegrade coronary blood flow, there has been much controversy concerning the indications for this procedure and the patch materials utilized during it. Additionally, the long-term results have rarely been reported, in contrast to the well-documented coronary artery bypass grafting (CABG) and percutaneous coronary intervention (PCI).

The aim of this retrospective study was to evaluate the long-term outcomes of SA-LMCA with a pericardial patch over 18 years.

## Methods

This study was approved by the investigational review board of Hallym University Kangdong Sacred Heart Hospital and an informed consent waiver was obtained. Between January 1995 and December 2002, SA-LMCA was performed in 24 consecutive patients with the left main coronary artery (LMCA) stenosis (mean age, 54.7 ± 11.7 years; 12 males and 12 females) at our institution. All operations were implemented by 2 surgeons (WYL and EJK) during their learning phase for coronary artery surgeries. LMCA stenosis involved the ostium or the proximal third in 15 patients (62.5%), the middle or distal third in 4 patients (16.7%), and the entire length in 5 patients (20.8%). Four patients received an intraaortic balloon pump (IABP) preoperatively and needed an emergency operation due to unstable hemodynamic conditions including one case with cardiac arrest. The main etiologic factor was atherosclerosis, although 5 patients were considered to have fibromuscular dysplasia. The preoperative characteristics of the study population are summarized in Table 1.

**Table 1:** **Preoperative patient characteristics**

Variable	Total number = 24 (%)
Hypertension	5 (21)
Diabetes	4 (17)
Dyslipidemia	7 (29)
Smoking	10 (42)
Previous cardiac surgery	1 (4)
Preoperative IABP	4 (17)
Emergency operation	4 (17)
Degree of left main coronary stenosis	
≥ 90%	10 (42)
50 ~ 89%	14 (58)
LVEF (mean ± SD)	59 ± 8%
Diagnosis	
Stable angina	4 (17)
Unstable angina	18 (75)
Myocardial infarction	2 (8)

IABP, Intraaortic balloon pump; LVEF, left ventricular ejection fraction; SD, standard deviation.

All operations were performed utilizing median sternotomy, with a mild hypothermic cardiopulmonary bypass and blood cardioplegia. Antegrade cardioplegic delivery was followed by retrograde infusion. The left ventricle was vented through the right superior pulmonary vein. The LMCA was approached anteriorly in all cases. The main pulmonary artery was divided in 12 patients (50.0%) and retracted to the left in 12 other patients. The incision began on the anterior wall of the aortic root, proceeded towards the LMCA, and crossed the stenosis. Onlay pericardial patches were continuously sewn from the distal LMCA to the aortic incision to obtain the funnel shape. A continuous 6–0 polypropylene suture was used for the LMCA segment, and a continuous 5–0 polypropylene suture was used for the aortic wall. The patch materials included fresh autologous pericardium in 7 patients and bovine pericardium in 17 patients at the surgeon’s discretion. Isolated SA-LMCA was performed in 16 (66.7%) patients. Among 8 other patients, this procedure was associated with 5 CABG, 1 mitral valve replacement, 1 repair of a partial atrioventricular septal defect, and 1 aortic valve replacement with surgical angioplasty of the right coronary ostium. Neither endarterectomy nor biopsy was performed in this series. The mean aortic cross clamp time was 102 ± 33 minutes, and the mean cardiopulmonary bypass time was 184 ± 61 minutes. None of the patients was given specific anticoagulation therapy, except for 200 mg of aspirin each day.

The definition of major adverse cardiac events (MACE) in this study included cardiac death, unexplained sudden death without an unequivocal noncardiac cause, myocardial infarction (MI), and repeat revascularization.

### Statistical analysis

We analyzed the overall survival, freedom from cardiac death, and freedom from MACE using the Kaplan-Meier method and a statistical software program (SPSS, version 13.0, Chicago, IL, USA).

## Results

There was no reversal to CABG due to technical failure of SA-LMCA, and there were no operative mortalities. The following major postoperative complications were observed: 2 cerebrovascular accidents (CVA), 1 case of mediastinitis, 1 case of bleeding requiring reoperation, and 1 perioperative MI with low cardiac output syndrome. It was difficult to wean the patient with perioperative MI from cardiopulmonary bypass. After undergoing a back-up CABG to the left anterior descending (LAD) coronary artery with placement of an IABP, he subsequently recovered from his perioperative MI, and demonstrated good patency of the LMCA and a graft to the LAD in a postoperative coronary angiography (CAG). All patients underwent a control CAG and exhibited a wide open and funnel-shaped LMCA. The patient characteristics, as well as the operative and follow-up data, are listed in Table 2.

**Table 2:** **Patient characteristics and, operative and follow-up data**

Pt	Age	Sex	Lesion site	Concomitant op	Patch	F/U (months)	Event (*) or cause of death	Results
1	47	F	O		B	227		A
2	55	F	E		B	194	PCI (179), sepsis	D
3	66	M	E	CABG-RCA	B	195	Cancer	D
4	60	M	D		A	223		A
5	62	M	M		B	222		A
6	35	F	O		A	220		A
7	56	M	D		B	133	CABG (132), LCOS^a^	D
8	43	M	O		A	214	Coronary spasm (5)	A
9	72	M	O	CABG-LAD	A	128	Traffic accident	D
10	55	M	E		B	206	CABG (111)	A
11	71	M	E	CABG-RCA	B	147	CVA	D
12	55	M	E		B	198		A
13	38	M	O		B	195		A
14	59	F	O	CABG-LAD	B	41	SCD	D
15	71	M	M		B	96	SCD	D
16	53	F	O		B	194		A
17	45	F	O		A	171		A
18	72	F	O	MVR	B	60	SCD	D
19	38	F	O		B	166		A
20	66	F	O	p-AVSD	B	166		A
21	54	F	O	AVR + **	A	158	PCI (79)	A
22	47	M	O	CABG-RCA	A	146	PCI (119)	A
23	57	F	O		B	133		A

Pt. patient; OP, operation; F/U, follow-up; Event (*), (time to MACE or to the last follow-up from the date of surgery, months); F, female; M, male; O, osteal or proximal; M, middle; D, distal; E, entire; CABG, coronary artery bypass grafting; RCA, right coronary artery; LAD, left anterior descending; MVR, mitral valve replacement; p-AVSD, repair of partial atrioventricular septal defect; AVR + **, aortic valve replacement + right coronary artery ostioplasty; B, bovine pericardium; A, autologous pericardium; PCI, Percutaneous coronary intervention; CVA, cerebrovascular accident; LCOS^a^, low cardiac output syndrome after a repeat CABG; SCD, sudden cardiac death; A; alive, D; death.

One patient was lost to follow-up (complete follow-up, 95.8%). The mean follow-up duration was 167 ± 51 months (range, 41 to 227 months). The patients in this study were followed up clinically and with echocardiography at our department during the early period and at the department of cardiology in our institution or a referring physician’s office in the later period. With the recurrence of chest pain, a repeat CAG was performed except in the patients who refused. During follow-up, there were 4 non-cardiac related deaths from a traffic accident (TA), CVA, sepsis, and lung cancer at 128, 147, 194 and 195 postoperative months, respectively. Three sudden cardiac deaths occurred at 41, 60, and 96 months.

A repeat CAG was performed in 12 (52.2%) patients at a mean interval of 93 postoperative months (range, 5–203 months) and revealed 4 cases of LMCA restenosis, 1 spasm of the LMCA, 1 distal right coronary artery (RCA) stenosis, and 6 wide open LMCA. Two of 4 patients with LMCA restenosis benefited from successful PCI at 79 and 119 months. Two other patients underwent repeat CABG at 111 and 132 months, with the second patient dying after the reoperation. The patient with RCA stenosis underwent a successful PCI at 179 months. In the 11 patients who were reluctant to undergo a repeat CAG, repeat echocardiographies revealed no interval changes during follow-up, but 3 patients suffered sudden cardiac deaths. Among 9 (39.1%) patients with MACE, 8 (34.8%) cases were related to the target lesion with the exception of one case with RCA stenosis and 4 of them died (3 sudden cardiac deaths and 1 death at reoperation). The onlay patch materials used in the 8 patients with target lesion related MACE were autologous pericardium in 3 cases (3/7, 42.9%) and bovine pericardium in 5 cases (5/16, 31.3%).

**Figure 1:**
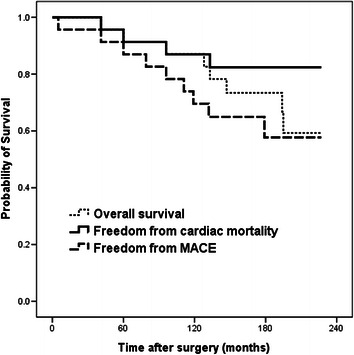
**Kaplan-Meier curves for overall survival of patients (n = 23), freedom from cardiac related mortality, and freedom from major adverse cardiac events (MACE).**

The cumulative estimates of the overall survival, freedom from cardiac death, and freedom from MACE at 5, 10, and 15 years via the Kaplan-Meier method are summarized as 95.7, 87.0, and 73.4%, respectively, 95.7, 87.0, and 82.4%, respectively, and 91.3, 69.6, and 57.7%, respectively. The survival curves are displayed in Figure 1.

## Discussion

Following its introduction by Effler and Sabiston in 1965, SA-LMCA was abandoned because of the high mortality and surgical failure. The excellent report by Hichcock and colleagues revived this procedure. Dion, Sullivan, Villemot, and coworkers published innovative reports concerning the surgical angioplasty approach and technique. We used the anterior approach exclusively and divided the main pulmonary artery for better visualization in half of our cases. This technique always provided an excellent view extending from the LMCA ostium to the bifurcation.

The indications for SA-LMCA are controversial, particularly regarding the extent and location of the LMCA stenosis and the existence of calcification. Botman and colleagues excluded patients with visible calcification and disease extending to the LMCA bifurcation [7]. The involvement of the distal LMCA or bifurcation may make it more difficult to reconstruct the LMCA and cause disastrous consequences.

Age is another issue related to the indications and surgical risks of this procedure. In this study, 5 of the 6 patients over 65 years of age died (2 cardiac and 3 non-cardiac deaths). Elderly patients may have more calcified lesions in the LMCA. Additionally, they often tend to have coexisting valvular disease, peripheral coronary artery disease, and comorbidities. Dion and colleagues gradually broadened their indications to include more distal stenosis, visible calcifications on CAG, advanced age, and decreased left ventricle (LV) function [8]. However, they concluded that SA-LMCA should be carefully attempted in patients with LMCA calcification or patients over 60 years of age. Most surgeons, including the authors of this study, agree with the opinions expressed by Dion regarding these indications.

The onlay patch material is the most important issue regarding the prevention of acute thrombosis and late restenosis of the LMCA. The saphenous vein and autologous pericardium have been commonly used for SA- LMCA [9]. The saphenous vein is well matched in size and preserves the fibrinolytic properties of the endothelium. However, its elasticity may cause a tendency to dilate. Dion and colleagues suggested that the saphenous vein might be preferable to autologous pericardium due to its potential fibrinolytic activity [4]. Martinovic and colleagues used a saphenous vein as the patch material in 27 patients and reported one aneurysmal dilatation [10]. In the present study, we used bovine pericardium in the majority of our patients (17/24) because of easy handling. We believe it would be more difficult to tailor and sew a saphenous vein or internal thoracic artery patch to the LMCA compared with a pericardial patch because of thinness and weakness. Currently, both the saphenous vein and bovine pericardium have been widely used in carotid endarterectomy (CEA) with patch angioplasty. Several studies have documented the safety, efficacy and durability of bovine pericardium as a CEA patch. In a previous study that analyzed 456 CEA cases over a 10-year period, both the carotid clamping time and total operation time were shorter in the bovine pericardial patch group compared with the saphenous vein patch group because of the easy handling and suturing. The study also revealed a similar incidence of restenosis between the two patch materials (2.8% for bovine pericardium versus 3.4% for saphenous vein) and identified 4 patients who developed late aneurysmal dilatation in saphenous vein patches compared with no cases involving bovine pericardial patches [11]. Another study also reported a low incidence of restenosis (1.6%, 4/256) over 12 years in patients who underwent CEA with bovine pericardial patch angioplasty [12]. Bovine pericardium also provides the benefit of off-the-shelf availability and, has a reliable consistency and strength to allow a tight fitting closure, which yields less suture line bleeding and prevents aneurysmal dilatation [13]. However, bovine pericardium also has disadvantage of incurring calcification, degeneration and restenosis.

In this series, we observed no acute or early thrombosis after SA-LMCA using a pericardial patch, despite the absence of specific anticoagulation therapy except for aspirin. However, the late failure of a pericardial patch caused 8 target lesion related MACE (5 in the bovine pericardial patch group and 3 the autologous pericardial patch group). Both pericardial patches exhibited similar MACE rates (bovine pericardium, 5/16 [31.3%]; autologous pericardium, 3/7 [42.9%]), although these numbers were too small to evaluate for the presence of statistically significant differences. Nevertheless, 4 patients in the bovine pericardial group died eventually at 41, 60, 96, and 132 postoperative months, while 3 patients in the autologous pericardial group survived a catastrophe. Notwithstanding the higher number of fatal consequences in the bovine pericardial group, we did not implement repeat CAG for the patients who suffered sudden cardiac death. Unfortunately, we have no information about the conditions of the LMCA with regard to mortalities. As restenosis is frequently asymptomatic, several authors recommend frequent imaging studies including surveillance CAG to avoid catastrophic consequences for patients undergoing SA-LMCA, regardless of whether they have cardiac symptoms [14]. It is currently unclear whether restenosis is directly related to the patch material, surgical techniques, or LMCA stenosis disease process per se. More data are necessary to establish standard guidelines regarding patch materials because of the small number of reported cases and short follow-up periods studied. In addition to commonly used patches, Liska and colleagues proposed using a proximal segment of the internal thoracic artery, and Malyshev et al. introduced the pulmonary autograft patch. Both studies reported excellent early results. The proximal right internal thoracic artery is sizable, pliable, and sufficiently robust to reconstruct the LMCA, in contrast with the distal segment. The pulmonary artery shares a common embryological origin with the aorta and has similar endothelial properties [15,16]. Internal thoracic artery and pulmonary autograft patches may be ideal patch materials and are preferable to the saphenous vein and pericardium, provided that they exhibit excellent long-term outcomes.

The cumulative estimates in this study at 5, 10, and 15 years are summarized as follows: overall survival of 96, 87, and 73%; freedom from cardiac death of 96, 87 and 82%; and freedom from MACE of 91, 70, and 58%, respectively. In a previous study evaluating a 15-year follow-up after CABG from the Coronary Artery Surgery Study (CASS) registry, the overall survival was 90, 74, and 56% at 5, 10, and 15 years, respectively [17]. Furthermore, another study from the CASS registry including 630 cases with left main equivalent coronary artery disease treated with CABG demonstrated a cumulative survival of 88, 69, and 44% at 5, 10, and 15 years, respectively [18]. Sabik and colleagues evaluated 3,803 patients treated with CABG for LMCA stenosis and reported an overall survival of 83, 64, and 44% at 5, 10, and 15 years, respectively [19]. Taggart and colleagues reviewed several studies of CABG and PCI for LMCA stenosis and reported an in-hospital mortality of 2-3% and a 30-day mortality of 3–4.2% after CABG for LMCA stenosis [14]. Considering that CABG is a well-established procedure in contrast with SA-LMCA, the early and long-term outcomes of this study are remarkable despite the small number of cases and great controversy surrounding patch materials.

Notwithstanding our important findings, the present study had a few limitations such as a retrospective observational design with a small sample size and the lack of a control group. Because of the small number of patients included in this study, we could not perform a multivariate statistical analysis or draw appropriate conclusions regarding statistical significance.

## Conclusions

SA-LMCA with a pericardial patch demonstrated acceptable late outcomes in selected patients with the LMCA stenosis, which were comparable to the well-documented CABG outcomes, despite the controversy regarding the indications and patch materials. Both pericardial patches exhibited similar MACE rates, whereas a bovine pericardial patch caused more fatal consequences.

## Abbreviations

SA-LMCA: Surgical angioplasty for left main coronary artery
LMCA: Left main coronary artery
MACE: Major adverse cardiac events
CABG: Coronary artery bypass graft
PCI: Percutaneous coronary intervention
IABP: Intraaortic balloon pump
MI: Myocardial infarction
CVA: Cerebrovascular accidents
LAD: Left anterior descending
CAG: Coronary artery angiography
TA: Traffic accident
RCA: Right coronary artery
LV: Left ventricle
CEA: Carotid endarterectomy
CASS: Coronary Artery Surgery Study
